# The Diagnostic Value of Exosome-Derived Biomarkers in Alzheimer's Disease and Mild Cognitive Impairment: A Meta-Analysis

**DOI:** 10.3389/fnagi.2021.637218

**Published:** 2021-03-01

**Authors:** Wenmin Xing, Wenyan Gao, Xiaoling Lv, Xiaogang Xu, Zhongshan Zhang, Jing Yan, Genxiang Mao, Zhibin Bu

**Affiliations:** ^1^Zhejiang Provincial Key Lab of Geriatrics, Department of Geriatrics, Zhejiang Hospital, Hangzhou, China; ^2^Key Laboratory of Neuropsychiatric Drug Research of Zhejiang Province, Institute of Materia Medica, Zhejiang Academy of Medical Sciences and Hangzhou Medical College, Hangzhou, China; ^3^Key Laboratory of Vector Biology and Pathogen Control of Zhejiang Province, Huzhou University, Huzhou, China; ^4^Huzhou Cent Hospital, Huzhou University, Huzhou, China

**Keywords:** Alzheimer's disease, mild cognitive impairment, exosomes, diagnosis, meta-analysis

## Abstract

**Background:** Alzheimer's disease (AD) diagnoses once depended on neuropathologic examination. Now, many widely used, validated biomarkers benefits for monitoring of AD neuropathologic changes. Exosome-derived biomarker studies have reported them to be significantly related to AD's early occurrence and development, although the findings are inconclusive. The aim of this meta-analysis was to identify exosome-derived biomarkers for the diagnosis of AD and mild cognitive impairment (MCI).

**Methods:** PubMed, PubMed Central, Web of Science, Embase, Google Scholar, Cochrane Library, the Chinese National Knowledge Infrastructure (CNKI), and the Chinese Biomedical Literature Database (CBM) were searched for studies assessing the diagnostic value of biomarkers, including data describing the pooled sensitivity (SEN), specificity (SPE), positive diagnostic likelihood ratio (DLR+), negative diagnostic likelihood ratio (DLR–), diagnostic odds ratio (DOR), and area under the curve (AUC). The quality of the included studies was assessed using RevMan 5.3 software. Publication bias was analyzed.

**Results:** In total, 19 eligible studies, including 3,742 patients, were selected for this meta-analysis. The SEN, SPE, DLR+, DLR–, DOR, and AUC (95% confidence intervals) of exosome-derived biomarkers in the diagnosis of AD or MCI were 0.83 (0.76–0.87), 0.82 (0.77–0.86), 4.53 (3.46–5.93), 0.21 (0.15–0.29), 17.27 (11.41–26.14), and 0.89 (0.86–0.92), respectively. Sub-group analyses revealed that studies based on serum or microRNA (miRNA) analysis, and those of Caucasian populations, AD patients, patient sample size >50, neuron-derived exosomes (NDE) from plasma and p-tau had higher sensitivity, specificity, and AUC values.

**Conclusion:** Exosome-derived biomarkers have shown potential diagnostic value in AD and MCI, although further research is required for confirmation.

## Introduction

Alzheimer's disease (AD), an age-associated neurodegenerative disorder, currently afflicts over 35.6 million individuals worldwide (Wortmann, 2012), while its prevalence continues to increase over time. The mortality rate of AD increased by 146.2% from 2000 to 2018, whereas deaths from human immunodeficiency virus (HIV) and heart disease decreased (Alzheimer's Association, 2020). Mild cognitive impairment (MCI), another type of neurodegenerative disorder, occurs in the stage between normal neurodegenerative aging and the development of AD, and such patients are more likely to develop dementia (Petersen et al., 2018). Some investigators have reported that about 45% of MCI patients remained in stable condition over time, whereas 28% developed AD and 15% recovered their cognitive function (Hu et al., 2017). It is generally understood that progressive neurodegeneration and the accumulation of amyloid β (Aβ) peptide and neurofibrillary tangles of tau proteins in the brain are the key characteristics of dementia (Rapoport et al., 2002). Changes in biomarkers involved in AD and MCI are closely related to the pathological mechanisms driving these conditions.

Abnormal change in the accumulation of Aβ peptides are currently used to identify the conversion from MCI to AD dementia and to distinguish AD patients from those with MCI or healthy individuals (Parnetti and Eusebi, 2018). A previous meta-analysis (Koychev et al., 2020) also showed that the levels of total tau (t-tau) and phosphorylated tau (p-tau) proteins in the cerebrospinal fluid (CSF) could significantly distinguish AD patients from healthy individuals. However, the discordances between positron emission tomography (PET) imaging and CSF biomarkers (CSF Aβ 42 vs. amyloid PET) had been described (Vos et al., 2016). Similarly, CSF t-tau just only reflected the intensity of dementia at a specific point, whereas elevated CSF p-tau represented an abnormal pathologic state depended by paired helical filament (PHF) tau formation (Blennow and Hampel, 2003). Moreover, Clifford R indicated that none of the biomarkers are as sensitive as direct examination of tissue at autopsy (Jack et al., 2018). In addition, widespread detection of these biomarkers in CSF has been impeded due to the invasiveness of the technique. Subsequently, blood became a more desirable target for isolating biomarkers to diagnose AD as an easier and less invasive means of collecting samples. Some studies have investigated t-tau and p-tau protein levels in the plasma of AD patients, although the results of these studies were deemed to be controversial (Tapiola et al., 2009; Chiu et al., 2014). Moreover, one previous meta-analysis also showed that plasma levels of Aβ42 were not a useful potential biomarker for the diagnosis of AD based on the analysis of more than 5,000 records (Olsson et al., 2016). Notably, in humans, carriers in the extracellular space transport a vast array of proteins or ribonucleic acids (RNAs), which remain protected against degradation by free ribonucleases (RNases) present in the blood (Van Niel et al., 2018). Exosomes are small membrane-bound vesicles, with a diameter of 30–150 nm, that contain functional molecules [proteins, microRNAs (miRNAs), and long non-coding RNAs (lncRNAs))] and other cellular components. Exosomes play important and diverse roles in various diseases by acting as barrier-permeable cellular carriers (Tapiola et al., 2009; Malm et al., 2016; Barile and Vassalli, 2017; Pegtel and Gould, 2019). The most recently available isolation kit and centrifuge used for the isolation of exosomes from plasma or serum is currently capable of ensuring the accurate quantification of exosomes or neurodegeneration-related proteins in exosomes. And it has been reported that Aβ42, t-tau, p-T181-tau, miRNAs, and other proteins in exosomes could distinguish AD or MCI patients from healthy individuals (Agliardi et al., 2019; Cha et al., 2019; Jia et al., 2019).

Although there is a growing body of research on exosomes related to AD or MCI, and the potential diagnostic value of exosomes in AD or MCI has been evaluated, the small number of cases included in each study has limited the diagnostic value of using them as potential AD or MCI clinical biomarkers. Additionally, a limitation of the 2011 NIA-AA (the National Institute on Aging and Alzheimer's Association) recommendations was that biomarkers were included just two categories- amyloid and tau-related neurodegeneration (Jack et al., 2018). In order to systematically illustrate the diagnostic value of multiple exosomal biomarkers in AD or MCI, we aimed to systematically review the published literature and perform a meta-analysis. Related data were extracted from the included studies and parameters describing the diagnostic value, such as the sensitivity (SEN) and specificity (SPE) were calculated, followed by subgroup analyses and an assessment of publication bias.

## Methods

The present meta-analysis was performed according to the Preferred Reporting Items for Systematic Reviews and Meta-analysis (PRISMA) guidelines (Moher et al., 2009).

### Search Strategy

Two investigators independently performed a literature search for articles in English or Chinese published before 31 September 2020 using databases that included PubMed, Web of Science, Google Scholar, Cochrane Library, the Chinese National Knowledge Infrastructure (CNKI), and the Chinese Biomedical Literature Database (CBM). The following key terms were used for the search: “Alzheimer's disease,” “mild cognitive impairment,” “exosomes,” “exosome,” “diagnosis,” “sensitivity,” “specificity,” and “ROC curve.” We also manually searched the relevant studies cited in the articles' references.

### Inclusion and Exclusion Criteria

Two authors independently read the titles and abstracts of the studies identified in the search of the databases. Searched articles were included in this meta-analysis according to the following criteria: (1) the diagnosis of AD or MCI was clinically confirmed according to the National Institute of Neurological and Communicative Disorders and Stroke and the Alzheimer's Disease and Related Disorders Association (NINCDS-ADRDA) criteria (Dubois et al., 2007); (2) the study included patients with AD or MCI, with healthy individuals as a control group; (3) the study assessed biological markers (for example, miRNAs or proteins) contained in exosomes; (4) exosome-derived markers for the diagnosis of AD or MCI were evaluated; (5) the study provided sufficient data about the diagnostic 2 × 2 tables; (6) the study was published in Chinese or English. Articles were excluded if they met any of the following criteria: (1) case reports or review articles; (2) duplicate articles; (3) articles not related to the assessment of diagnostic value; and (4) articles not related to exosomes.

### Data Extraction and Quality Assessment

Two authors independently extracted the following data from each of the included studies: the first author, publication year, region from which the study population was derived, type of sample, the source, content, and isolation method of the exosomes, the number of case and control groups, and the true-positive (TP), false-positive (FP), true-negative (TN), and false-negative (FN) numbers. Any inconsistency was resolved by a third researcher.

The quality of the included studies was independently assessed by two of the authors using the Quality Assessment of Diagnostic Accuracy Studies 2 (QUADAS-2) tool (Whiting et al., 2011).

### Statistical Analysis

Statistical analyses were performed using Stata 12.0 (Stata Corporation, College Station, TX, USA), RevMan 5.3 (https://community.cochrane.org/help/tools-and-software/revman-5). Diagnostic parameters, including the SEN, SPE, positive diagnostic likelihood ratio (DLR+), negative diagnostic likelihood ratio (DLR-), and diagnostic odds ratio (DOR) were calculated using a bivariate random effects regression model (Reitsma et al., 2005). The summary receiver operator characteristic (SROCs) curves were calculated, along with the pooled area under the curve (AUC) values with the corresponding 95% confidence intervals (CIs) (Hamza et al., 2009). Heterogeneity among the studies was evaluated using the *Q* test and based on the *I*^2^ statistic, with significant heterogeneity between studies defined as an *I*^2^ > 50% (Higgins et al., 2003). To further explore the heterogeneity, subgroup analysis and meta-regression were performed using Stata 12.0 software. Publication bias was assessed by generating a Deeks' funnel plot. Lastly, the post-test probability was evaluated by drawing Fagan's nomogram. *P-*values < 0.05 were considered to be indicative of statistically significant differences.

## Results

### Characteristics of the Included Studies and Individuals

**Figure 1:**
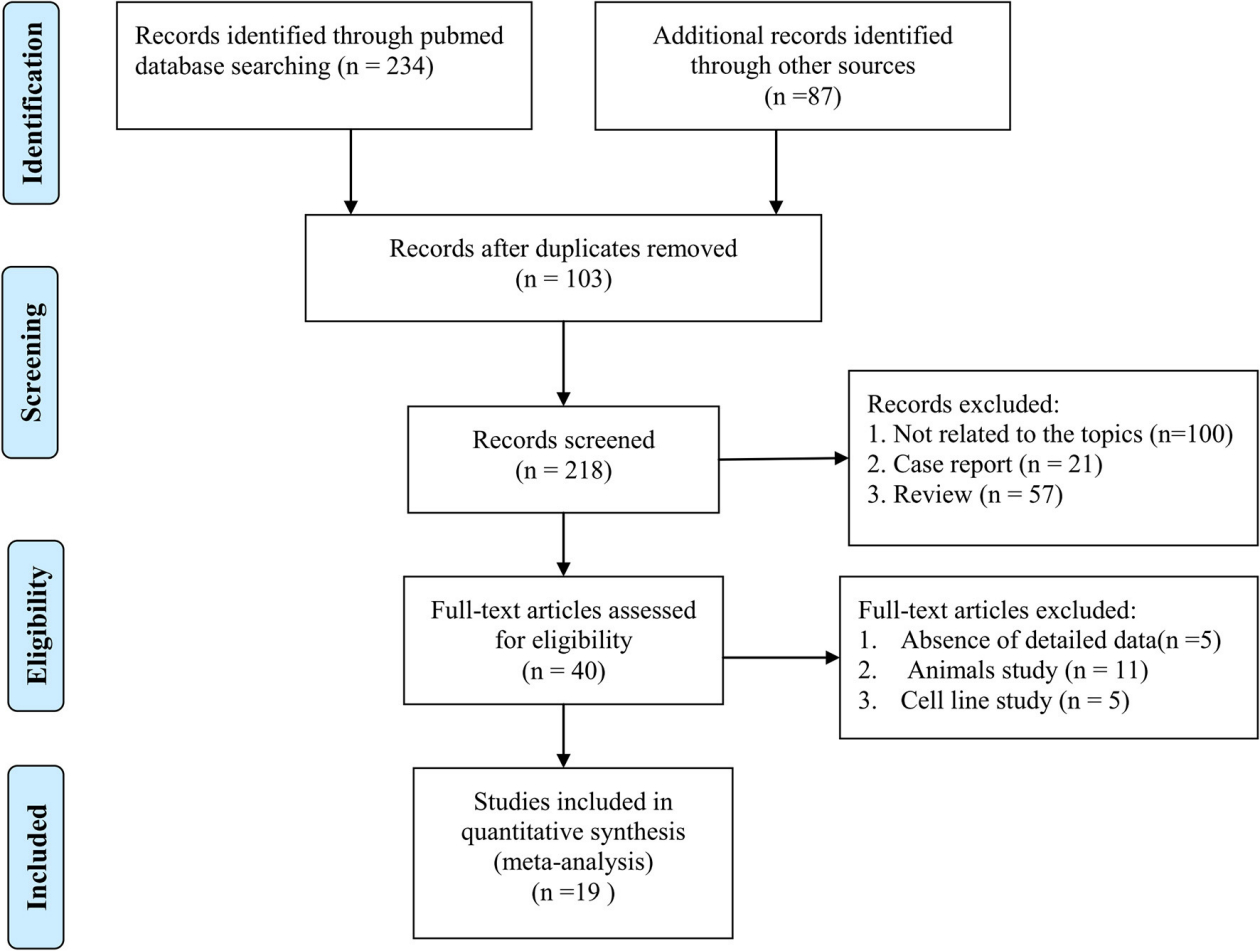
Flowchart diagram of selecting studies process.

According to the search results, a total of 321 articles were retrieved from the databases, among which, 103 duplicated articles were removed. We then removed 178 articles that did not meet the inclusion criteria based on the abstracts. Based on the full-text versions of the articles, we removed another 21 articles that did not meet our inclusion and exclusion criteria either due to a lack of detailed data to allow the calculation of TP, FP, TN, or FN values or a lack of a healthy control group as a comparator. Finally, we retrieved 19 articles that met all the criteria (Fiandaca et al., 2015; Goetzl et al., 2015, 2016; Kapogiannis et al., 2015; Winston et al., 2016, 2018; Wei et al., 2018; Yang et al., 2018; Agliardi et al., 2019; Cha et al., 2019; Fotuhi and Khalaj-Kondori, 2019; Jia et al., 2019, 2020; Barbagallo et al., 2020; Gu et al., 2020; Nam and Lee, 2020; Perrotte et al., 2020; Wang et al., 2020; Zhao et al., 2020). The detailed study selection process is shown in the flow diagram (Figure 1).

A summary of the characteristics of the 19 included studies is shown in Table 1; overall, the studies included 3,742 individuals (1,587 AD patients, 334 MCI patients, and 1,821 healthy individuals). The included studies were published from 2014 to 2020 and were conducted in Asia (Wei et al., 2018; Yang et al., 2018; Jia et al., 2019, 2020; Gu et al., 2020; Nam and Lee, 2020; Wang et al., 2020; Zhao et al., 2020), North America (Fiandaca et al., 2015; Goetzl et al., 2015, 2016; Kapogiannis et al., 2015; Winston et al., 2016, 2018; Perrotte et al., 2020) and Europe (Agliardi et al., 2019; Cha et al., 2019; Fotuhi and Khalaj-Kondori, 2019; Barbagallo et al., 2020). The exosome sources included plasma and serum samples. Enzyme-linked immunosorbent assays (ELISAs) were used to detect the expression of protein markers (e.g., t-tau protein and Aβ42 protein), and quantitative reverse transcription polymerase chain reaction (qRT-PCR) was used to detect the expression level of miRNAs (Wei et al., 2018; Yang et al., 2018; Cha et al., 2019; Barbagallo et al., 2020). The sample sizes of the included studies ranged from 12 to 100, and all AD or MCI patients were diagnosed based on Mini-Mental State Examination (MMSE) scores and clinical histopathologic examinations. The healthy control individuals had regular MMSE scores. Additionally, the publication languages were limited to English and Chinese.

**Table 1:** Characteristics of subjects and the included studies in this meta-analysis.

**Author, year**	**Country**	**Study group**	**No**.	**Sex** **(female /%)**	**Age (SD/median)**	**MMSE (SD/median)**	**Sample source**	**Exosome isolation method**	**Marker analytical method**	**Disease markers**	**QUADAS-2 score**
Jia et al. (2020)	China	AD	73	42 (57.5)	65 (6)	19.6 (3.1)	Blood plasma	Isolation kit	ELISA	GAP43	3
										SNAP25	
		MCI	71	39 (54.9)	66 (7)	26.2 (0.4)				Neurogranin	
										Synaptotagmin 1	
		HC	72	37 (51.4)	64 (5)	29.3 (1.2)					
Jia et al. (2019)	China	AD	73	42 (57.5)	65 (6)	19.6 (3.1)	Blood plasma	Isolation kit	ELISA	Aβ42	3
										t-Tau	
		MCI	71	39 (54.9)	66 (7)	26.2 (0.4)				p-T181-tau	
		HC	72	37 (51.4)	64 (5)	29.3 (1.2)					
Goetzl et al. (2016)	USA	AD	12	13 (62)	74.4 ± 6.84	26.3 ± 3.45	Neuron-derived exosomes from plasma	Isolation kit	ELISA	Synaptophysin	2
										Synaptopodin	
		HC	12	13 (62)	74.4 ± 6.84	29.8 ± 0.39				Synaptotagmin-2	
		MCI	16	4 (25)	63.6 6 1.82	19.7 6 2.57					
										Neurogranin	
Winston et al. (2018)	USA	MCI	31	63.6(–)	70.2 ± 2.3	27.9 ± 0.64	Neuron-derived exosomes	Isolation kit	ELISA	Aβ42	2
		HC	36	61.5(–)	67.8 ± 2.3	29.1 ± 0.33					
Fotuhi and Khalaj-Kondori (2019)	Iran	AD	16	31(–)	76.4 ± 7.89	19.33 ± 5.0	Blood plasma	Isolation kit	PCR	lncRNA BACE1-AS	2
		HC	36	25(–)	79.7 ± 8.16	27.30 ± 0.54					
Zhao et al. (2020)	China	MCI	87	47 (54%)	66.2 (4.3)	25.7 (1.4)	Neuron-derived exosomes from plasma	Isolation kit	ELISA	Aβ42	2
										SS16	
		AD	88	50 (47%)	67.7 (4.2)	17.0 (2.1)					
		HC	80	44 (55%)	67.3 (4.7)	29.3 (0.7)					
Nam and Lee (2020)	Korea	MCI	29	12(–)	75.13 ± 0.99	23.17 ± 0.20	Neuron-derived exosomes	Isolation kit	ELISA	t-tau	3
										p-tau	
		AD	18	3(–)	76.55 ± 1.33	16.55 ± 0.52					
		HC	23	17(–)	73.92 ± 0.88	27.69 ± 0.16					
Yang et al. (2018)	China	AD	100	66(–)	74.15	-	Serum exosome	Isolation kit	PCR	miR-135a	1
										miR-193b	
										miR-384	
		HC	100	(–)	-	-					
Winston et al. (2016)	USA	AD	10	(–)	-	-	Neuron-derived exosomes from plasma	Isolation kit	ELISA	p-T181-tau	2
										p-S396-	
		MCI	20	7	68.70 ± 7.76	28.95 ± 0.26				tauAβ1-42	
		ADC	20	9	75.35 ± 6.82	27.35 ± 0.29					
		HC	10	(–)	-	-					
Fiandaca et al. (2015)	USA	AD	57	27(–)	79.5 ± 6.05	-	Neuron-derived blood exosomes	Isolation kit	ELISA	p-T181-tau	2
										t-tau	
										Aβ1-42	
		HC	57	27(–)	79.5 ± 6.03	-					
Goetzl et al. (2015)	USA	AD	24	12(–)	75.7 ± 7.59	-	Neuron-derived exosomes	Isolation kit	ELISA	LRP6	2
		HC	24	12(–)	75.1 ± 7.18	-				HSF1	
										REST	
Kapogiannis et al. (2015)	USA	AD	26	13(–)	74.3 ± 7.48	-	Neuron-derived exosomes from plasma	Isolation kit	ELISA	Total IRS-1	1
										p-serine	
		HC	26	13(–)	74.3 ± 7.48	-				312-IRS-1	
										p-Pan-tyrosine-IRS-1	
Dong et al. (2020)	China	AD	31	(–)	68.58 ± 8.04	15.93 ± 6.61	Neuron-derived exosomes from plasma	Isolation kit	ELISA	Aβ42	2
										p-T181-tau	
										MMP-9	
		HC	15	(–)	64.80 ± 6.00	27.67 ± 1.72					
Wang et al. (2020)	China	AD	68	(–)	73.7 ± 7.7	13.7 ± 6.7	Blood plasma	Isolation kit	PCR	BACE 1-A S	3
		HC	55	(–)	71.8 ± 8.1	27.2 ± 2.1					
Perrotte et al. (2020)	Canada	AD	36	29(–)	79,1 ± 1.1	19.90 ± 1.39	Blood plasma	Isolation kit	ELISA	t-Tau	2
										APP level	
										p-Tau-T181	
		MCI	12	11(–)	75.33 ± 1.19	27.90 ± 0.31					
		HC	12	9(–)	68.8 ± 1.5	29.42 ± 0.29					
Cha et al. (2019)	German	AD	31	(–)	70–105	-	Neuron-derived exosomes from plasma	Ultracentrifugation	PCR	mir132	2
		MCI	16	(–)	55–85	-				mir212	
		HC	16	(–)	85–105	-					
Barbagallo et al. (2020)	Italy	AD	30	16(–)	72.6 ± 8.1	13.1 ± 5.7	Serum	Isolation kit	PCR	miR-22, miR-23a, miR-29a, miR-125b	2
		HC	30	20(–)	67.9 ± 8.2	-					
Agliardi et al. (2019)	Italy	AD	24	16(–)	77.67 ± 1.40	21.91 ± 0.91	Serum	Isolation kit	Western blot	SNAP-25	1
		HC	17	13(–)	76.47 ± 1.49	28.73 ± 0.43					
Wei et al. (2018)	China	AD	32	13	79.3 ± 8.9	27.2 ± 1.3	Serum	Isolation kit	PCR	miR-223	2
		HC	16	8	79.5 ± 6.8	13 ± 4.2					

*AD, Alzheimer's disease; MCI, mild cognitive impairment; HC, healthy controls; MMSE, Mini-Mental State Examination; CSF, cerebrospinal fluid; ELISA, enzyme-linked immunosorbent assay; GAP43, growth associated protein 43; neurogranin, SNAP2, synaptosome associated protein 25; QUADAS-2, Quality Assessment of Diagnosis Accuracy Studies*.

The quality of all the included studies was assessed using the QUADAS-2 tool, most of which obtained high scores on the QUADAS-2 assessments. All are summarized in Table 1.

### Diagnostic Performance

**Figure 2:**
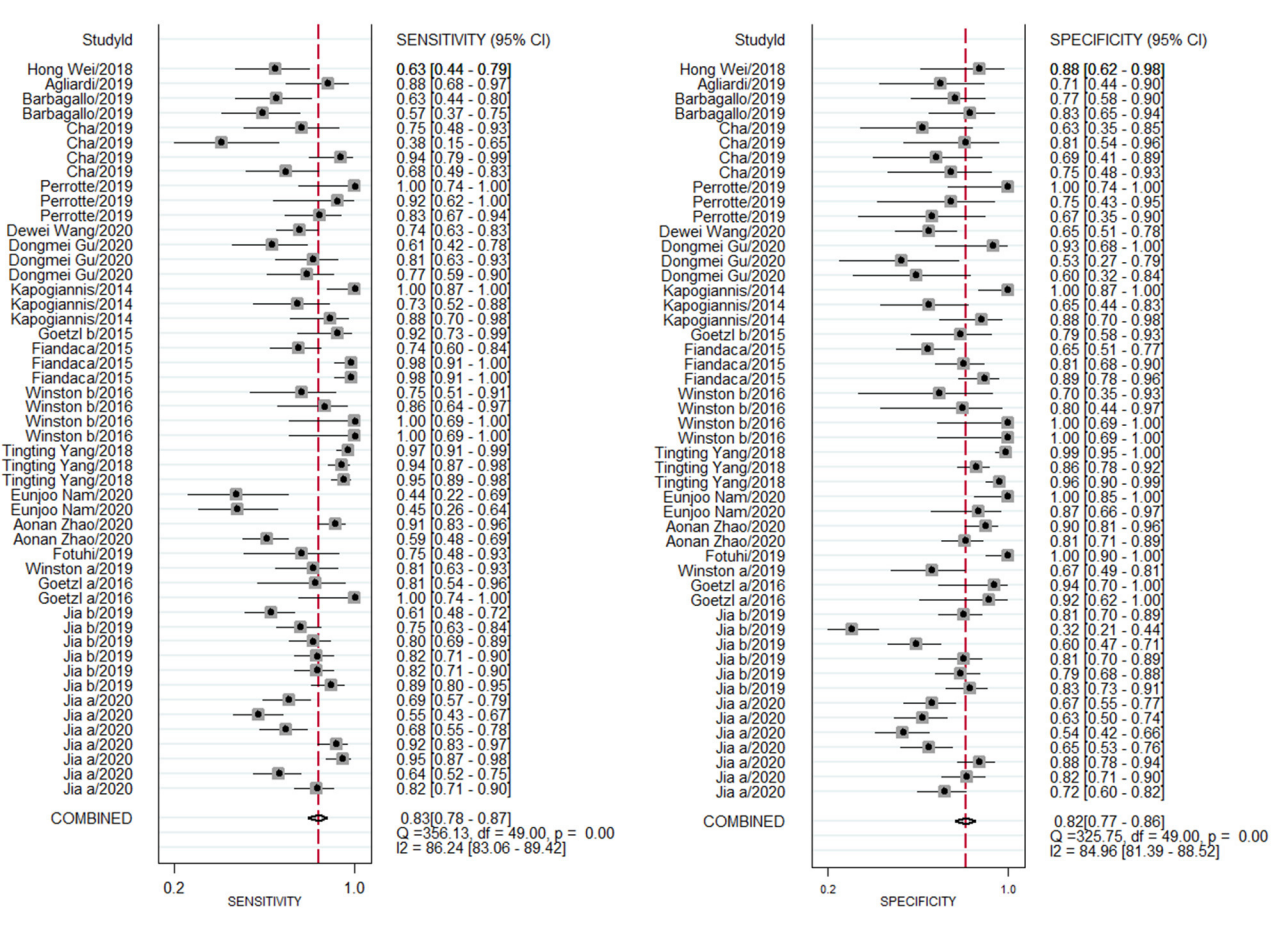
Forest plot of sensitivity and specificity of exosome-drived biomarkers for the diagnosis of Alzheimer's disease (AD) and mild cognitive impairment (MCI).

**Figure 3:**
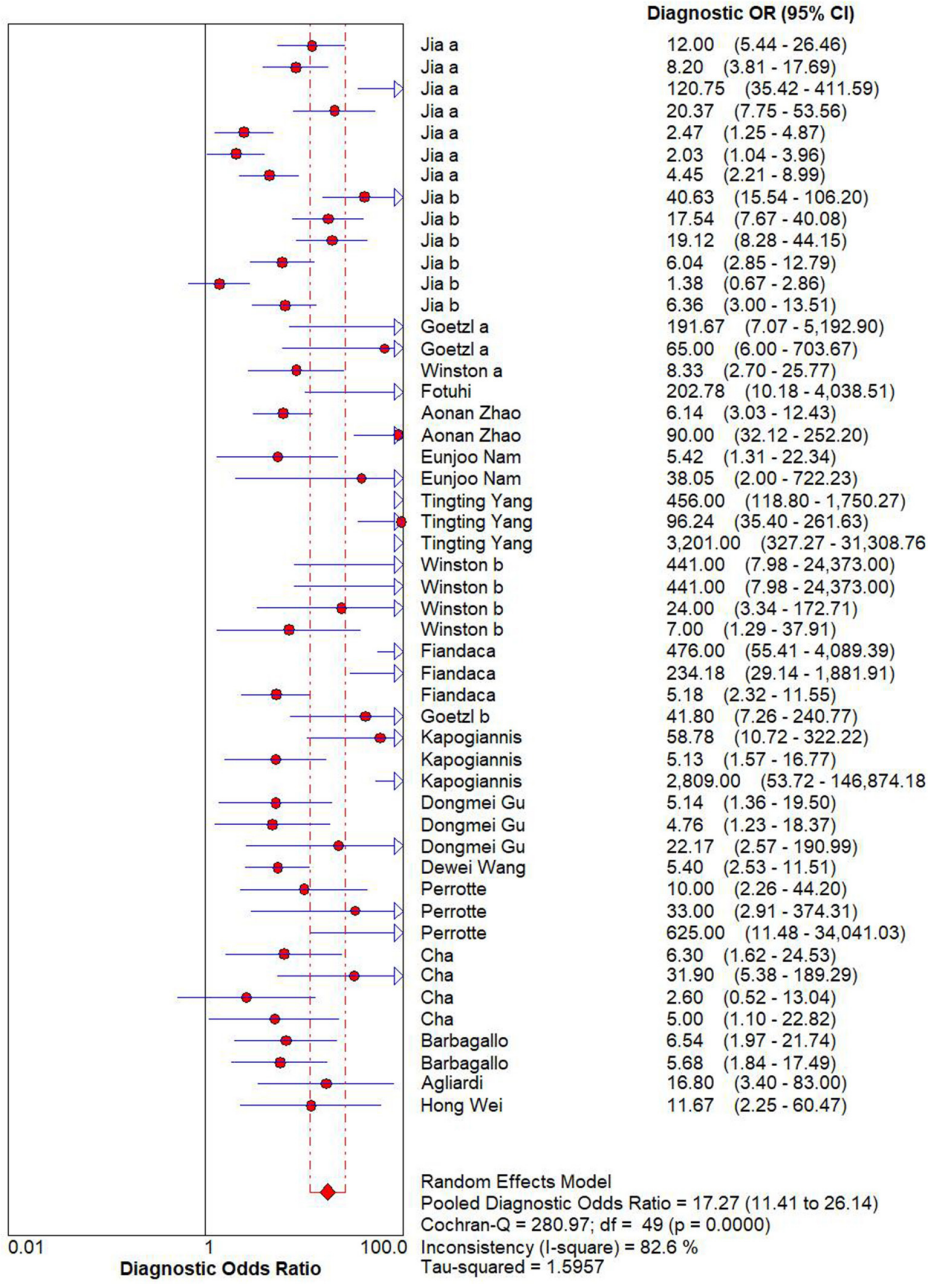
Forest plot of DLR + and DLR– of exosome-drived biomarkers for the diagnosis of Alzheimer's disease (AD) and mild cognitive impairment (MCI).

**Figure 4:**
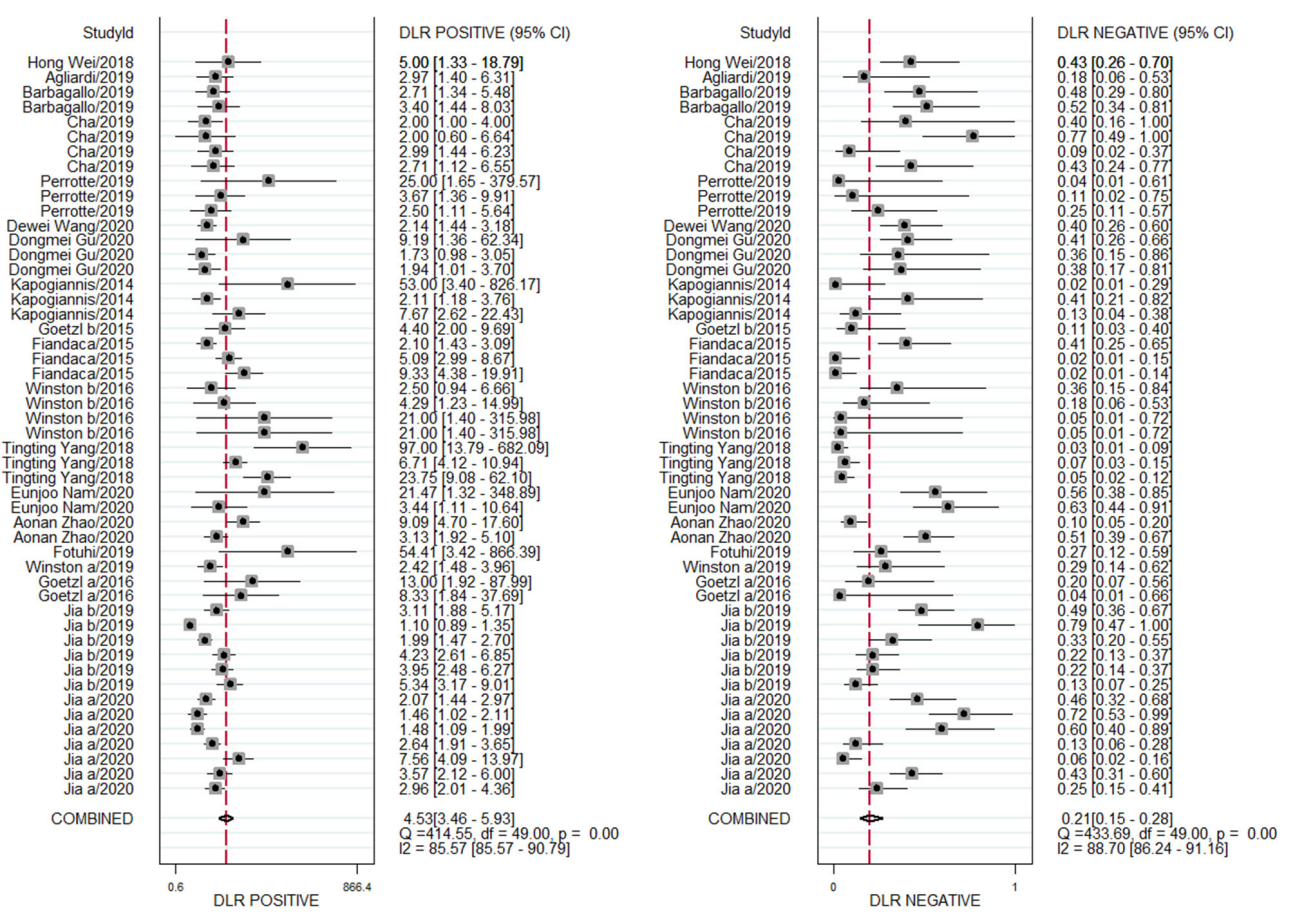
Forest plot of DOR of exosome-drived biomarkers for the diagnosis of Alzheimer's disease (AD) and mild cognitive impairment (MCI).

**Figure 5:**
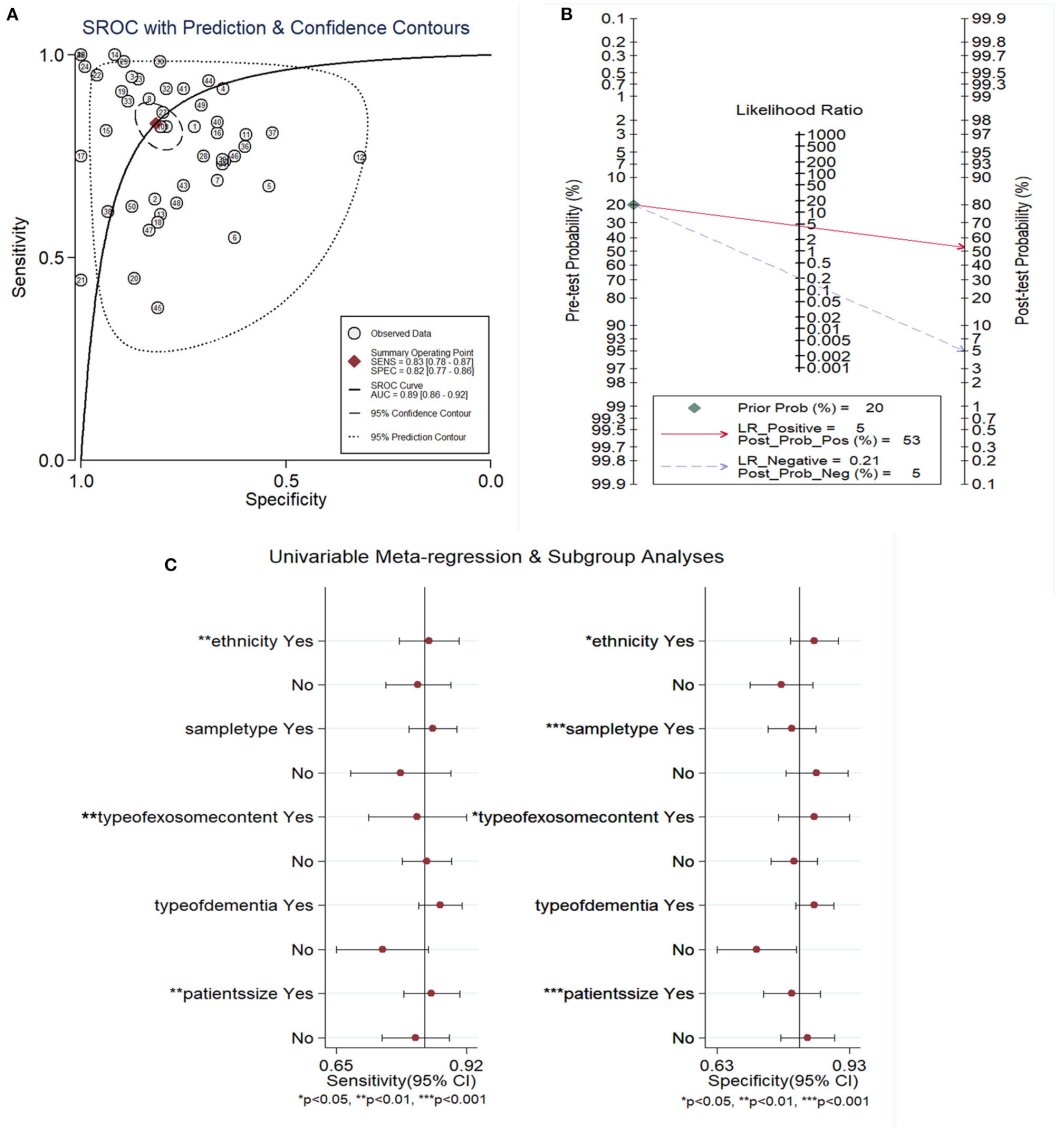
The SROC **(A)** Fagan's nomogram assessing **(B)** and forest plot of covariates' meta-regression **(C)** of exosome-drived biomarkers for the diagnosis of AD and MCI. A: AUC, area under the curve; SENS, sensitivity; SPEC, specificity; SROC, summary receiver operator characteristic; AD, Alzheimer's disease; MCI, mild cognitive impairment.

A random effects model was used to evaluate the pooled diagnostic effect. The pooled results for the SEN and SPE are shown in Figure 2. The pooled SEN of the 19 included studies was 0.83 (95% CI: 0.78–0.87, *I*^2^= 86.24%, *P* < 0.01) and the pooled SPE was 0.82 (95% CI: 0.77–0.86, *I*^2^= 84.96%, *P* < 0.01). The pooled results for the DLR+ and DLR- are shown in Figure 3. The pooled DLR+ and DLR- were 4.53 (95% CI: 3.46–5.93, *I*^2^ = 85.57%, *P* < 0.01; Figure 4) and 0.21 (95% CI: 0.15–0.29, *I*^2^ = 88.70%, *P* < 0.01; Figure 4), respectively. The DOR value was 17.27 (95% CI: 11.41–26.14, *I*^2^= 82.60%, *P* < 0.01; Figure 3), and the AUC was 0.89 (95% CI: 0.86–0.92; Figure 5A).

The above findings demonstrated that exosome-derived biomarkers had a high potential diagnostic value for AD or MCI. However, the Cochran's Q values for the SEN and SPE were 356.13 and 325.75, respectively, and the *I*^2^ values for the SEN and SPE were 86.24 and 84.96%, respectively, suggesting significant heterogeneity in the analysis. Additional analysis needed to be performed to explore the source(s) of this heterogeneity.

### Meta-Regression and Subgroup Analysis

Meta-regression was performed to explore the source of the potential heterogeneity. The ethnicity (Caucasian or not), sample type (plasma or not), type of exosome content (miRNA or not), sample size (>50 or not), and type of dementia (AD or not) were used as covariates to conduct the meta-regression analysis. As shown in Figure 5C, the ethnicity, type of exosomal content, and sample size were likely to be the sources of the heterogeneity in the SEN, whereas ethnicity, sample type, type of exosomal content, and sample size all had *P*-values <0.05, <0.01, or <0.001, making them the likely sources of the heterogeneity seen in the SPE.

Next, the subgroup analysis was performed in the following characteristics: ethnicity, sample type, type of exosome content, sample size, exosome sources and type of exosome protein (Aβ42, t-tau, plasma p-tau). As shown in Table 2, exosomal markers had higher diagnostic ability in the Caucasian than the Asian population; for example, higher SEN (0.85 vs. 0.82) SPE (0.85 vs. 0.78), DLR+ (5.5 vs. 3.7), DOR (31 vs. 16), and AUC (0.91 vs. 0.87) values were observed in the Caucasian population. In the analysis of the exosome source (sample type), exosomes isolated from serum exhibited high SEN (0.86), SPE (0.89), DOR (48), and AUC (0.94) values. For the subgroup based on exosome contents, we found significant differences between the protein and miRNA groups; for example, the SPE (0.85 vs. 0.81), DLR+ (5.4 vs. 4.3), and DOR (25 vs. 21) values were higher in the miRNA subgroup compared to the protein subgroup, which may indicate that exosome-derived miRNAs could be used to more efficiently discriminate between AD or MCI patients and healthy individuals. Moreover, the differences in exosomal markers were also assessed between the AD and MCI patients based on data extracted from 19 studies. That comparison showed that the SEN (0.87 vs. 0.73), SPE (0.85 vs. 0.71), DLR+ (5.9 vs. 2.6), DOR (37 vs. 7), and AUC (0.92 vs. 0.78) values of AD patients were all higher than those of individuals with MCI. Compared to studies with a sample size <50, studies with sample sizes of 50 or higher exhibited higher SEN values (0.81 vs. 0.76), but a lower SPE (0.83 vs. 0.80) in dementia patients. In addition, compared with plasma total exosome group, NDE isolated from plasma obtained significant higher SEN (0.85 vs. 0.80), SPE (0.83 vs. 0.75), DLR+ (5.0 vs. 3.2), AUC (0.90 vs. 0.85). Lastly, the diagnostic value of Aβ42, t-tau and p-tau was also evaluated, and p-tau exhibited the optimal diagnostic value with higher AUC (0.91), SEN (0.85), SPE (0.85), and DOR (31), and following with Aβ42 (SEN: 0.86, SPE: 0.78, AUC: 0.88) and t-tau (SEN: 0.78, SPE: 0.75, AUC: 0.83).

**Table 2:** Results of subgroup analysis in diagnostic meta-analysis.

**Subgroups**	**Number of studies**	**SEN (95% CI)**	**SPE (95% CI)**	**DLR+ (95% CI)**	**DLR- (95% CI)**	**DOR (95% CI)**	**AUC**
**Ethnicity**							
Caucasian	28	0.85 (0.77–0.91)	0.85 (0.79–0.89)	5.5 (3.8–7.9)	0.18 (0.11–0.29)	31(15–65)	0.91
Asian	22	0.82 (0.75–0.87)	0.78 (0.70–0.84)	3.7 (2.5–5.4)	0.24 (0.16–0.35)	16 (8–32)	0.87
**Sample type**							
Plasma	43	0.82 (0.77–0.87)	0.80 (0.74–0.84)	4.1 (3.1–5.3)	0.22 (0.16–0.30)	18 (11–31)	0.88
Serum	7	0.86 (0.70–0.94)	0.89 (0.78–0.95)	7.8 (3.5–17.5)	0.16 (0.07–0.39)	48 (10–242)	0.94
**Type of exosome content**							
Protein	39	0.83 (0.78–0.88)	0.81 (0.75–0.85)	4.3 (3.2–5.7)	0.21 (0.15–0.29)	21 (12–36)	0.89
miRNA	11	0.82 (0.68–0.91)	0.85 (0.75–0.91)	5.4 (2.9–9.9)	0.22 (0.11–0.43)	25 (7–83)	0.90
**Type of dementia**							
AD	33	0.87 (0.81–0.91)	0.85 (0.80–0.89)	5.9 (4.2–8.1)	0.16 (0.11–0.23)	37 (20–71)	0.92
MCI	17	0.73 (0.65–0.80)	0.71 (0.63–0.79)	2.6 (1.9–3.4)	0.38 0.28–0.51)	7 (4–12)	0.78
**Patient size**							
<50	27	0.81 (0.73–0.87)	0.83 (0.76–0.88)	4.7 (3.3–6.7)	0.23 (0.15–0.34)	21 (11–40)	0.89
>50	23	0.84 (0.78–0.89)	0.80 (0.73–0.86)	4.3 (2.9–6.3)	0.19 (0.13–0.29)	22 (10–47)	0.89
**Exosome source**							
Plasma total exosome	18	0.80 (0.74–0.85)	0.75 (0.66–0.82)	3.2 (2.3–4.5)	0.27 (0.19–0.37)	12 (6–23)	0.85
NDE from plasma	25	0.85 (0.76–0.91)	0.83 (0.77–0.88)	5.0 (3.5–7.1)	0.18 (0.11–0.31)	27 (13–58)	0.90
Serum total exosome	7	0.86 (0.70–0.94)	0.89 (0.78–0.95)	7.8 (3.5–17.5)	0.16 (0.07–0.39)	48 (10–242)	0.94
**Type of exosome protein**							
Aβ42	9	0.86 (0.75–0.92)	0.78 (0.69–0.85)	4.0 (2.6–6.0)	0.18 (0.10–0.34)	22 (8–58)	0.88
T-tau	6	0.78 (0.63–0.87)	0.75 (0.51–0.89)	3.1 (1.3–7.0)	0.30 (0.16–0.57)	10 (3–40)	0.83
P-tau	8	0.85 (0.68–0.94)	0.85 (0.74–0.92)	5.5 (3.1–10.0)	0.18 (0.08–0.41)	31 (10–103)	0.91

*NDE, neuronally derived exosomes*.

### Clinical Diagnostic Value of Exosome-Derived Biomarkers in AD and MCI

To evaluate the diagnostic value of exosome-derived markers in AD and MCI, a Fagan nomogram was constructed. As shown in Figure 5B, when there was low suspicion of AD or MCI (20%), the post-test probability for a positive test was 53%. The LR- was 0.21, which decreased the post-test probability to 5% for a negative test.

### Publication bias

An analysis of publication bias was also performed. Deeks' funnel plot asymmetry test showed that there was no publication bias (Supplementary Figure 1).

## Discussion

Numerous studies have confirmed that exosome-derived proteins, lncRNAs or miRNAs can be stably detected in body fluids (He et al., 2018). These molecules have been considered as novel biomarkers for the diagnosis of neurodegenerative diseases, including AD (Dong et al., 2020); however, the potential diagnostic value of quantifying exosome-derived biomarkers for AD or MCI has not previously been confirmed through a systematic analysis. Thus, this meta-analysis was performed to elucidate the diagnostic value of exosome-derived biomarkers in AD or MCI. Nineteen eligible studies were included in the present meta-analysis. The results indicated that exosome-derived biomarkers might serve as valuable cognitive biomarkers for AD or MCI diagnoses.

Overall, the pooled diagnostic SEN, SPE, and AUC were determined to be 0.83, 0.82, and 0.89, respectively. Exosome-derived markers exhibited a performance for distinguishing AD and MCI patients in Caucasian populations than in Asian populations, given an AUC of 0.91 vs. an AUC of 0.87, respectively. In serum samples, exosome-derived markers had a higher diagnostic value for AD and MCI diagnoses compared with exosome-derived markers in plasma, with a higher AUC of 0.94. Hence, exosome-derived markers isolated from serum might be a more accurate and non-invasive detection method. In addition, exosome-derived markers seemed to distinguish AD from healthy individuals with more power than they could distinguish MCI patients from healthy individuals (AUCs of 0.92 vs. 0.78, respectively). Aβ and tau protein, which could be packaged inside exosomes, aggregated in the brain, and then transported into CSF and blood (Gu et al., 2020). So we compared the diagnostic value between plasma total exosomes and NDE-drived from plasma. Sure enough, NDE-drived from plasma presented more potential diagnosis than plasma total exosomes. Interestingly, although Aβ and tau protein had been guided as the gold standard for diagnosis AD, p-tau showed the best potential diagnosis value for AD in this meta-analysis, which was worth to be confirmed in the further large scale studies.

As cellular membranes (diameter: 30–100 nm) secreted by certain cell-types, exosomes can be isolated from bodily fluids. Neurodegenerative disease-associated proteins or miRNAs, such as Aβ1-42, tau, p-tau, and miRNA-22 are secreted in exosomes during their formation (Jia et al., 2019; Barbagallo et al., 2020). Interestingly, exosomes may readily penetrate the blood-brain barrier (BBB) and spread throughout the brain *via* synaptic delivery (Andjus et al., 2020) as a result of their small size and cell membrane-like structure. Previous studies have also shown that intravenously injected exosomes can move across the BBB and transfer biological molecules into neurons (Alvarez-Erviti et al., 2011). Moreover, exosomes can more easily carry Aβ peptides and tau proteins into the blood across the BBB under pathological conditions, in addition to growth-associated protein 43 (GAP43), synaptosomal-associated protein 25 (SNAP25), neurogranin, synaptotagmin-1, miR-135a, miR-193b, and miR-384. A previous study also reported that Aβ42, T-tau, and P-T181-tau derived from exosomes in the blood could accurately diagnose AD and predict its occurrence up to ten years before its clinical onset; these findings were also confirmed via detection in CSF (Jia et al., 2019).

However, there was an important limitation in this meta-analysis. The protein biomarkers detection method in included studies was only by ELISA method. During the detection, adequate protection from heterophilic antibodies and other blood molecules that might interfere in the measurement, which couldn't ensure analytical sensitivity and specificity (Zetterberg and Blennow, 2020). But, ultrasensitive assays, for example Simoa (single molecule array) and LC-MS method, could reduce the risk of molecular interference and avoid the combination with heterophilic antibodies in the sample diluent, which had obtained a reliable quantification. Neurofilament light (NfL), one neurodegeneration biomarker, was firstly quantified using Simoa assay technology. And later, plasma Aβ40, Aβ42 were detected by the same method (Zetterberg and Bendlin, 2021). And Karikari also developed a very sensitive and specific p-tau181 assay for plasma and serum samples by using a sandwich immunoassay format on Simoa (Karikari et al., 2020). Although there were no included studies to detect AD biomarkers by using Simoa assay, fortunately, NDE biomarkers of phosphorylated tau and insulin receptor substrate 1 were validated with Simoa assay in cognitively normal participants who developed AD (Kapogiannis et al., 2019).

Some other limitations should also be considered in this comprehensive and systematic meta-analysis. Firstly, further studies with larger populations are needed to confirm these results. Secondly, the samples in the included studies were tested at different time points, which could be problematic, as miRNAs in the blood could have been altered in cases of prolonged storage times. Thirdly, the results of some included studies had not been verified by the additional assessment of biomarkers in the CSF, which could have led to some of the measurements being inaccurate. Lastly, the levels of some proteins, such as p-s396-tau, were relatively low. The ELISA-based method used for protein quantification may contribute to a low SEN and SPE, which also limits the interpretability of this meta-analysis.

In conclusion, we found that exosome-derived biomarkers had high diagnostic value for AD and MCI. The sample type, type of exosomal content, and sample size all impacted the biomarkers' diagnostic value in AD and MCI. However, the present results could not distinguish between different stages of AD and MCI based solely on biomarker expression levels. Further studies are needed to confirm the relationship between biomarker expression levels and the different stages of AD and MCI. In the future, it will be possible to construct a detailed system based on exosome-derived biomarkers for the diagnosis of AD and MCI that could lead to earlier detection and intervention.

## Data Availability Statement

The raw data supporting the conclusions of this article will be made available by the authors, without undue reservation.

## Author Contributions

WX and WG performed the literature review, conducted data analysis, and manuscript preparation. XL and ZZ performed the literature review and manuscript preparation. WG and XX helped in the literature review and data analysis. ZB, GM, and JY conducted literature review, designed the study, and performed data analysis and manuscript preparation. All authors contributed to the article and approved the submitted version.

## Conflict of Interest

The authors declare that the research was conducted in the absence of any commercial or financial relationships that could be construed as a potential conflict of interest.

## References

[B1] Agliardi C. Guerini F. R. Zanzottera M. Bianchi A. Nemni R. Clerici M. (2019). SNAP-25 in serum is carried by exosomes of neuronal origin and is a potential biomarker of Alzheimer's disease. Mol. Neurobiol. 56, 5792–5798. 10.1007/s12035-019-1501-x 30680692

[B2] Alvarez-Erviti L. Seow Y. Yin H. Betts C. Lakhal S. Wood M. J. (2011). Delivery of siRNA to the mouse brain by systemic injection of targeted exosomes. Nat. Biotechnol. 29, 341–345. 10.1038/nbt.1807 21423189

[B3] Alzheimer's Association (2020). 2020 Alzheimer's disease facts and figures. Alzheimers Dement. 2, 459–509. 10.1016/j.jalz.2016.03.001 27570871

[B4] Andjus P. Kosanović M. Milićević K. Gautam M. Vainio S. J. Jagečić D.. (2020). Extracellular vesicles as innovative tool for diagnosis, regeneration and protection against neurological damage. Int. J. Mol. Sci. 21:6859. 10.3390/ijms21186859 32962107 PMC7555813

[B5] Barbagallo C. Mostile G. Baglieri G. Giunta F. Luca A. Raciti L.. (2020). Specific signatures of serum miRNAs as potential biomarkers to discriminate clinically similar neurodegenerative and vascular-related diseases. Cell. Mol. Neurobiol. 40, 531–546. 10.1007/s10571-019-00751-y 31691877 PMC11448951

[B6] Barile L. Vassalli G. (2017). Exosomes: therapy delivery tools and biomarkers of diseases. Pharmacol. Ther. 174, 63–78. 10.1016/j.pharmthera.2017.02.020 28202367

[B7] Blennow K. Hampel H. (2003). CSF markers for incipient Alzheimer's disease. Lancet Neurol. 2, 605–613. 10.1016/S1474-4422(03)00530-1 14505582

[B8] Cha D. J. Mengel D. Mustapic M. Liu W. Selkoe D. J. Kapogiannis D.. (2019). miR-212 and miR-132 are downregulated in neurally derived plasma exosomes of Alzheimer's patients. Front. Neurosci. 13:1208. 10.3389/fnins.2019.01208 31849573 PMC6902042

[B9] Chiu M. J. Chen Y. F. Chen T. F. Yang S. Y. Yang F. P. Tseng T. W.. (2014). Plasma tau as a window to the brain-negative associations with brain volume and memory function in mild cognitive impairment and early Alzheimer's disease. Hum. Brain Mapp. 35, 3132–3142. 10.1002/hbm.22390 24129926 PMC6869439

[B10] Dong X. Zheng D. Nao J. (2020). Circulating exosome microRNAs as diagnostic biomarkers of dementia. Front. Aging Neurosci. 12:580199. 10.3389/fnagi.2020.580199 33093831 PMC7506134

[B11] Dubois B. Feldman H. H. Jacova C. Dekosky S. T. Barberger-Gateau P. Cummings J.. (2007). Research criteria for the diagnosis of Alzheimer's disease: revising the NINCDS-ADRDA criteria. Lancet Neurol. 6, 734–746. 10.1016/S1474-4422(07)70178-3 17616482

[B12] Fiandaca M. S. Kapogiannis D. Mapstone M. Boxer A. Eitan E. Schwartz J. B.. (2015). Identification of preclinical Alzheimer's disease by a profile of pathogenic proteins in neurally derived blood exosomes: a case-control study. Alzheimers Dement. 11, 600–607.e601. 10.1016/j.jalz.2014.06.008 25130657 PMC4329112

[B13] Fotuhi S. N. Khalaj-Kondori M. (2019). Long non-coding RNA BACE1-AS may serve as an Alzheimer's disease blood-based biomarker. Int. J. Mol. Sci. 69, 351–359. 10.1007/s12031-019-01364-2 31264051

[B14] Goetzl E. J. Boxer A. Schwartz J. B. Abner E. L. Petersen R. C. Miller B. L.. (2015). Low neural exosomal levels of cellular survival factors in Alzheimer's disease. Ann. Clin. Transl. Neurol. 2, 769–773. 10.1002/acn3.211 26273689 PMC4531059

[B15] Goetzl E. J. Kapogiannis D. Schwartz J. B. Lobach I. V. Goetzl L. Abner E. L.. (2016). Decreased synaptic proteins in neuronal exosomes of frontotemporal dementia and Alzheimer's disease. FASEB J. 30, 4141–4148. 10.1096/fj.201600816R 27601437 PMC5102122

[B16] Gu D. Liu F. Meng M. Zhang L. Gordon M. L. Wang Y.. (2020). Elevated matrix metalloproteinase-9 levels in neuronal extracellular vesicles in Alzheimer's disease. Ann. Clin. Transl. Neurol. 7, 1681–1691. 10.1002/acn3.51155 32790155 PMC7480907

[B17] Hamza T. H. Arends L. R. Van Houwelingen H. C. Stijnen T. (2009). Multivariate random effects meta-analysis of diagnostic tests with multiple thresholds. BMC Med. Res. Methodol. 9:73. 10.1186/1471-2288-9-73 19903336 PMC2787531

[B18] He C. Zheng S. Luo Y. Wang B. (2018). Exosome Theranostics: biology and translational medicine. Theranostics 8, 237–255. 10.7150/thno.21945 29290805 PMC5743472

[B19] Higgins J. P. Thompson S. G. Deeks J. J. Altman D. G. (2003). Measuring inconsistency in meta-analyses. BMJ 327, 557–560. 10.1136/bmj.327.7414.557 12958120 PMC192859

[B20] Hu C. Yu D. Sun X. Zhang M. Wang L. Qin H. (2017). The prevalence and progression of mild cognitive impairment among clinic and community populations: a systematic review and meta-analysis. Int. Psychogeriatr. 29, 1595–1608. 10.1017/S1041610217000473 28884657

[B21] Jack C. R. Jr. Bennett D. A. Blennow K. Carrillo M. C. Dunn B. Haeberlein S. B.. (2018). NIA-AA research framework: toward a biological definition of Alzheimer's disease. Alzheimers Dement. 14, 535–562. 10.1016/j.jalz.2018.02.018 29653606 PMC5958625

[B22] Jia L. Qiu Q. Zhang H. Chu L. Du Y. Zhang J.. (2019). Concordance between the assessment of Aβ42, T-tau, and P-T181-tau in peripheral blood neuronal-derived exosomes and cerebrospinal fluid. Alzheimers Dement. 15, 1071–1080. 10.1016/j.jalz.2019.05.002 31422798

[B23] Jia L. Zhu M. Kong C. Pang Y. Zhang H. Qiu Q.. (2020). Blood neuro-exosomal synaptic proteins predict Alzheimer's disease at the asymptomatic stage. Alzheimers Dement. 17, 49–60. 10.1002/alz.12166 32776690 PMC7984076

[B24] Kapogiannis D. Boxer A. Schwartz J. B. Abner E. L. Biragyn A. Masharani U.. (2015). Dysfunctionally phosphorylated type 1 insulin receptor substrate in neural-derived blood exosomes of preclinical Alzheimer's disease. FASEB J. 29, 589–596. 10.1096/fj.14-262048 25342129 PMC4314222

[B25] Kapogiannis D. Mustapic M. Shardell M. D. Berkowitz S. T. Diehl T. C. Spangler R. D.. (2019). Association of extracellular vesicle biomarkers with Alzheimer disease in the baltimore longitudinal study of aging. JAMA Neurol. 76, 1340–1351. 10.1001/jamaneurol.2019.2462 31305918 PMC6632160

[B26] Karikari T. K. Pascoal T. A. Ashton N. J. Janelidze S. Benedet A. L. Rodriguez J. L.. (2020). Blood phosphorylated tau 181 as a biomarker for Alzheimer's disease: a diagnostic performance and prediction modelling study using data from four prospective cohorts. Lancet. Neurol. 19, 422–433. 10.1016/S1474-4422(20)30071-5 32333900

[B27] Koychev I. Jansen K. Dette A. Shi L. Holling H. (2020). Blood-based ATN biomarkers of Alzheimer's disease: a meta-analysis. J. Alzheimers Dis. 79, 177–195. 10.3233/JAD-200900 33252080

[B28] Malm T. Loppi S. Kanninen K. M. (2016). Exosomes in Alzheimer's disease. Neurochem. Int. 97, 193–199. 10.1016/j.neuint.2016.04.011 27131734

[B29] Moher D. Liberati A. Tetzlaff J. Altman D. G. (2009). Preferred reporting items for systematic reviews and meta-analyses: the PRISMA statement. PLoS Med. 6:e1000097. 10.1371/journal.pmed.1000097 19621072 PMC2707599

[B30] Nam E. Lee Y. B. (2020). Serum tau proteins as potential biomarkers for the assessment of Alzheimer's disease *Progression* 21:5007. 10.3390/ijms21145007 32679907 PMC7404390

[B31] Olsson B. Lautner R. Andreasson U. Öhrfelt A. Portelius E. Bjerke M.. (2016). CSF and blood biomarkers for the diagnosis of Alzheimer's disease: a systematic review and meta-analysis. Lancet Neurol. 15, 673–684. 10.1016/S1474-4422(16)00070-3 27068280

[B32] Parnetti L. Eusebi P. (2018). Cerebrospinal fluid biomarkers in Alzheimer's disease: an invaluable tool for clinical diagnosis and trial enrichment. J. Alzheimers Dis. 64, S281–s287. 10.3233/JAD-179910 29562517

[B33] Pegtel D. M. Gould S. J. (2019). Exosomes. Annu. Rev. Biochem. 88, 487–514. 10.1146/annurev-biochem-013118-111902 31220978

[B34] Perrotte M. Haddad M. Le Page A. Frost E. H. Fulöp T. Ramassamy C. (2020). Profile of pathogenic proteins in total circulating extracellular vesicles in mild cognitive impairment and during the progression of Alzheimer's disease. Neurobiol. Aging 86, 102–111. 10.1016/j.neurobiolaging.2019.10.010 31883770

[B35] Petersen R. C. Lopez O. Armstrong M. J. Getchius T. S. D. Ganguli M. Gloss D.. (2018). Practice guideline update summary: mild cognitive impairment: report of the guideline development, dissemination, and implementation subcommittee of the American Academy of Neurology. Neurology 90, 126–135. 10.1212/WNL.0000000000004826 29282327 PMC5772157

[B36] Rapoport M. Dawson H. N. Binder L. I. Vitek M. P. Ferreira A. (2002). Tau is essential to beta -amyloid-induced neurotoxicity. Proc. Natl. Acad. Sci. U.S.A. 99, 6364–6369. 10.1073/pnas.092136199 11959919 PMC122954

[B37] Reitsma J. B. Glas A. S. Rutjes A. W. Scholten R. J. Bossuyt P. M. Zwinderman A. H. (2005). Bivariate analysis of sensitivity and specificity produces informative summary measures in diagnostic reviews. J. Clin. Epidemiol. 58, 982–990. 10.1016/j.jclinepi.2005.02.022 16168343

[B38] Tapiola T. Alafuzoff I. Herukka S. K. Parkkinen L. Hartikainen P. Soininen H.. (2009). Cerebrospinal fluid {beta}-amyloid 42 and tau proteins as biomarkers of Alzheimer-type pathologic changes in the brain. Arch. Neurol. 66, 382–389. 10.1001/archneurol.2008.596 19273758

[B39] Van Niel G. D'angelo G. Raposo G. (2018). Shedding light on the cell biology of extracellular vesicles. Nat. Rev. Mol. Cell Biol. 19, 213–228. 10.1038/nrm.2017.125 29339798

[B40] Vos S. J. B. Gordon B. A. Su Y. Visser P. J. Holtzman D. M. Morris J. C.. (2016). NIA-AA staging of preclinical Alzheimer disease: discordance and concordance of CSF and imaging biomarkers. Neurobiol. Aging 44, 1–8. 10.1016/j.neurobiolaging.2016.03.025 27318129 PMC4913039

[B41] Wang D. Wang P. Bian X. Xu S. Zhou Q. Zhang Y.. (2020). Elevated plasma levels of exosomal BACE1-AS combined with the volume and thickness of the right entorhinal cortex may serve as a biomarker for the detection of Alzheimer's disease. Mol. Med. Rep. 22, 227–238. 10.3892/mmr.2020.11118 32377715 PMC7248487

[B42] Wei H. Xu Y. Xu W. Zhou Q. Chen Q. Yang M.. (2018). Serum exosomal miR-223 serves as a potential diagnostic and prognostic biomarker for dementia. Neuroscience 379, 167–176. 10.1016/j.neuroscience.2018.03.016 29559383

[B43] Whiting P. F. Rutjes A. W. Westwood M. E. Mallett S. Deeks J. J. Reitsma J. B.. (2011). QUADAS-2: a revised tool for the quality assessment of diagnostic accuracy studies. Ann. Intern. Med. 155, 529–536. 10.7326/0003-4819-155-8-201110180-00009 22007046

[B44] Winston C. N. Goetzl E. J. Akers J. C. Carter B. S. Rockenstein E. M. Galasko D.. (2016). Prediction of conversion from mild cognitive impairment to dementia with neuronally derived blood exosome protein profile. Ann. Clin. Transl. Neurol. 3, 63–72. 10.1016/j.dadm.2016.04.001 27408937 PMC4925777

[B45] Winston C. N. Goetzl E. J. Baker L. D. Vitiello M. V. Rissman R. A. (2018). Growth hormone-releasing hormone modulation of neuronal exosome biomarkers in mild cognitive impairment. J. Alzheimers Dis. 66, 971–981. 10.3233/JAD-180302 30372675 PMC6487872

[B46] Wortmann M. (2012). Dementia: a global health priority—highlights from an ADI and World Health Organization report. Alzheimers Res. Ther. 4:40. 10.1186/alzrt143 22995353 PMC3580397

[B47] Yang T. T. Liu C. G. Gao S. C. Zhang Y. Wang P. C. (2018). The serum exosome derived MicroRNA-135a,−193b, and−384 were potential Alzheimer's disease biomarkers. Biomed. Environ. Sci. 31, 87–96. 10.3967/bes2018.011 29606187

[B48] Zetterberg H. Bendlin B. B. (2021). Biomarkers for Alzheimer's disease-preparing for a new era of disease-modifying therapies. Mol. Psychiatry 26, 296–308. 10.1038/s41380-020-0721-9 32251378 PMC8172244

[B49] Zetterberg H. Blennow K. (2020). Blood biomarkers: democratizing Alzheimer's diagnostics. Neuron 106, 881–883. 10.1016/j.neuron.2020.06.004 32553204

[B50] Zhao A. Li Y. Yan Y. Qiu Y. Li B. Xu W.. (2020). Increased prediction value of biomarker combinations for the conversion of mild cognitive impairment to Alzheimer's dementia. Transl. Neurodegener. 9:30. 10.1186/s40035-020-00210-5 32741361 PMC7397685

