# Supplementary figures and images for: The Diagnostic Value of Exosome-Derived Biomarkers in Alzheimer's Disease and Mild Cognitive Impairment: A Meta-Analysis
Source: Front Aging Neurosci. 2021 Mar 1;13:637218. doi: 10.3389/fnagi.2021.637218 (PMC7957006; doi:10.3389/fnagi.2021.637218)

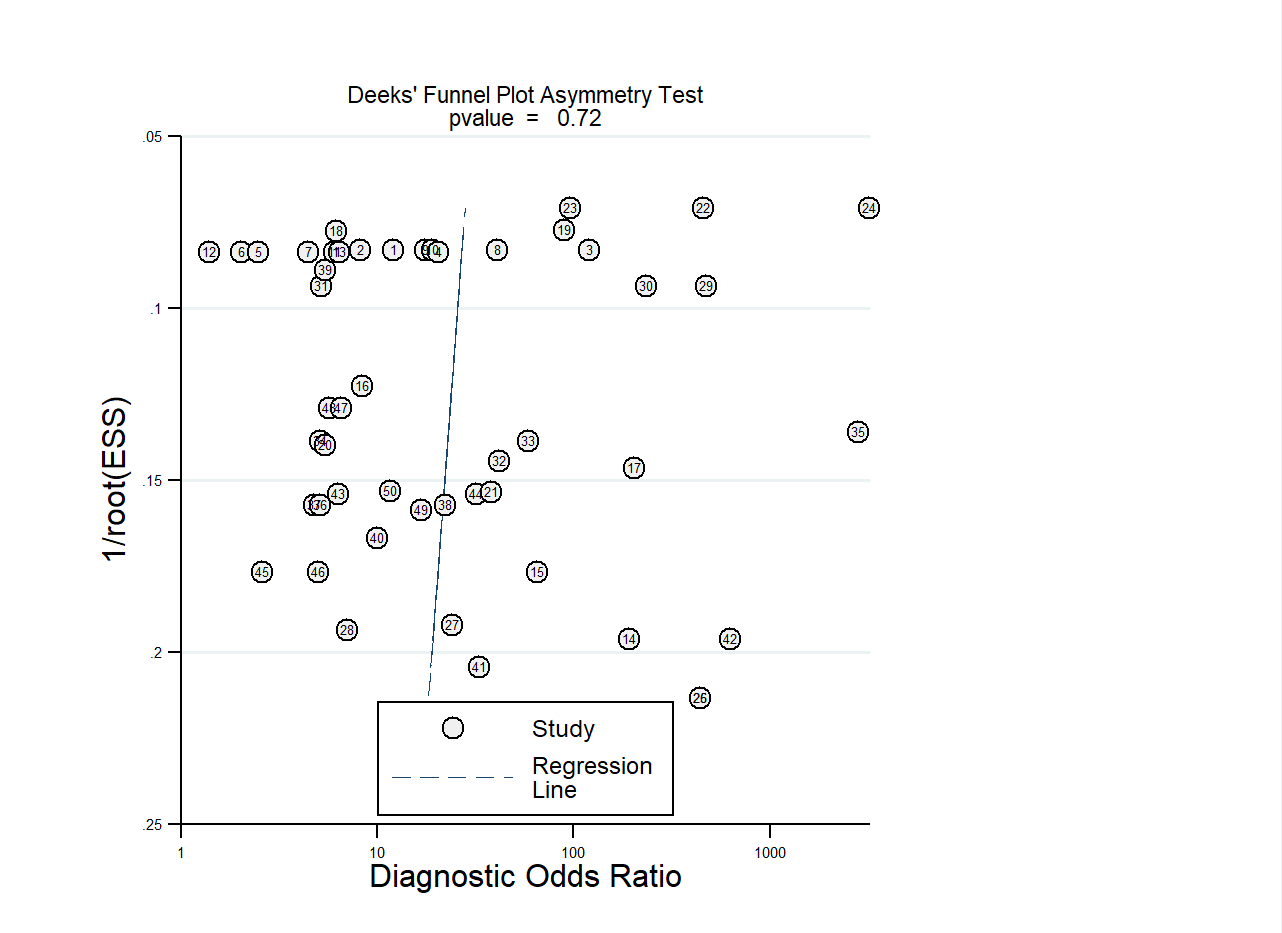

Supplement: Supplementary file 1 [file Image_1.TIF]
